# Ultraviolet Radiation Promoted Hypoxia-Induced Apoptosis in HL-60 Human Promyelocytic Leukemia Cell Line

**DOI:** 10.1155/2022/7702481

**Published:** 2022-10-31

**Authors:** Yilin Wang, Jiandong Sun, Dong Xie, Ren Zhong, Shaoyong Si, Xiaotong Liu, Zhenghai Qu, Lirong Sun, Lingzhen Wang

**Affiliations:** ^1^Department of Pediatric Hematology, The Affiliated Hospital of Qingdao University, Qingdao, Shandong 266003, China; ^2^Pediatric Intensive Care Department, The Affiliated Hospital of Qingdao University, Qingdao, Shandong 266003, China; ^3^Department of Thoracic Surgery, The Affiliated Hospital of Qingdao University, Qingdao, Shandong 266003, China; ^4^Department of Pediatrics, The Affiliated Hospital of Qingdao University, Qingdao, Shandong 266003, China

## Abstract

Minimal residual disease (MRD) is an important reason for the failure of autologous hematopoietic stem cell transplantation (auto-HSCT). Reducing MRD in grafts is particularly important to improve the efficacy of auto-HSCT. Previously, we reported that ultraviolet light-emitting diode (UV LED) suppressed the expression of Bcl-2 to induce apoptosis in HL-60 cells. Leukemia can lead to severe hypoxia of the bone marrow. Therefore, this study aimed to investigate the effect of UV LED on leukemia cells under hypoxia. HL-60 cells were irradiated with a UV LED (30 J/m^2^) and simulated under hypoxia with cobalt chloride. We found that UV LED irradiation or CoCl_2_ inhibited proliferation, induced apoptosis, decreased the Bcl-2/Bax ratio, and increased the levels of caspase 3, cleaved-caspase 3, and caspase 9 in HL-60 cells. In particular, the combined application of UV and CoCl_2_ significantly enhanced the apoptosis of HL-60 cells. In conclusion, UV LED in hypoxia exacerbated the inhibition of proliferation and induction of apoptosis and necrosis in HL-60 cells via the regulation of caspase 3/9 and the Bcl-2/Bax ratio-dependent pathway. The application of UV LEDs in hypoxia conditions may be a promising approach to kill residual drug-resistant leukemia cells in autologous grafts.

## 1. Introduction

Leukemia is the most common hematological malignancy in children and with the widespread use of chemical drugs, the prognosis of some children with leukemia has been significantly improved. At present, the 5-year overall survival (OS) of acute lymphocytic leukemia (ALL) patients has improved to approximately 90%. The OS of acute myeloid leukemia (AML) has increased to approximately 60% [1]. However, 10–15% of ALL patients and nearly half of AML patients still require autologous hematopoietic stem cell transplantation (auto-HSCT) for further treatment.

Auto-HSCT was originally used as an alternative treatment for leukemia patients who did not have a suitable donor for allogeneic hematopoietic stem cell transplantation (allo-HSCT). For hematopoietic recovery or hematopoietic reconstruction, auto-HSCT can produce a better antitumor effect than chemotherapy and avoid the side effects of repeated chemotherapy such as organ damage and multidrug resistance [2].

Peripheral blood stem cells (PBSCs) have become the preferred source of stem cells for auto-HSCT, and they are usually collected in the early stages of chemotherapy [3, 4]. However, at this time, autologous hematopoietic stem cell transplants tend to contain residual cells that are resistant to chemotherapy, which is known as minimal residual disease (MRD). This condition can easily lead to transplantation failure and recurrence after transplantation. Using various purification methods to reduce tumor cell contamination in transplants and increase the sensitivity of residual tumor cells in transplants to chemotherapeutic drugs has become an important measure to improve the efficacy of auto-HSCT.

Hypoxia is a common feature of the tumor microenvironment, and it could inhibit tumor cell proliferation and induce apoptosis by affecting the expression of hypoxia-induciblefactor-1*α* (HIF-1*α*) and the production of oxygen species (ROS) [5]. In in vitro studies, hypoxia is usually induced by reducing the atmospheric oxygen concentration or by using chemicals such as cobalt chloride (CoCl_2_) and deferoxamine (DFO) [6, 7].

Ultraviolet (UV), especially in the medium wave range (UVB, 280–320 nm), is an important environmental factor affecting human health. Paradoxically, UV-induced immunosuppression may have the therapeutic potential [8]. Clinically, UV has been used to treat skin diseases and tumors. UV is a powerful inducer of apoptosis by inducing DNA damage, reactivation of death receptors, and production of reactive ROS. Ultraviolet light-emitting diodes (LEDs) can replace traditional ultraviolet lamps in the fields of sterilization, water purification, and medical treatment [9]. Previously, we reported that irradiation with 30 J/m^2^ of a 280 nm UV LED inhibited the proliferation of HL-60 cells and induced apoptosis and necrosis in vitro [10]. However, the effect of 280 nm UV LED irradiation on cells under hypoxia is not clear. In this study, we investigated the effect of 280 nm UV LED irradiation on HL-60 human leukemia cells cultured under hypoxia. Our results showed that the combination of hypoxia and ultraviolet irradiation could significantly inhibit the viability of HL-60 human leukemia cells.

## 2. Materials and Methods

### 2.1. Cell Culture and Treatment

Promyelocytic leukemia HL-60 cells were provided by the American Type Culture Collection (Manassas, VA, USA) and cultured in Iscove's modified Dulbecco's medium (BI, Logan, UT, USA) containing 10% fetal bovine serum (BI) and 1% penicillin (BI) at 37°C in a 5% CO_2_ incubator. Cells in logarithmic growth were selected for the experiments and were divided into the control (without treatment), UV LED (cells were irradiated with 280 nm UV LED (Qingdao Ziyuan Photoelectronic Co., Ltd., Qingdao, China) at a dose of 30 J/m^2^)), CoCl_2_ (cells were treated with 150 *μ*М CoCl_2_ for 48 h), UV + CoCl_2_ (cells were pretreated with 280 nm UV LED at a dose of 30 J/m^2^ and then treated with 150 *μ*М CoCl_2_ for 48 h), 2UV (cells were irradiated once, and then, irradiated again at the 24th hour), and 2UV + CoCl_2_ groups (cells were treated with 150 *μ*М CoCl_2_ for 48 h, during which they were irradiated with UV LED twice at an interval of 24 h).

The lamp head of the LED UV lamp corresponds to the size of one single well of a 24-well plate. HL-60 cells were resuspended with culture medium after centrifugation and seeded in a 24-well plate at a density of 1 × 10^6^ cells/well. The cells were incubated for 1 h and irradiated after the cells formed a single-cell suspension. Irradiation dose (J/m^2^) = irradiation intensity (W/m^2^) × time(s). Referring to previous studies, the best irradiation dose was 30 J/M^2^. The intensity of the UV lamp was measured and adjusted every time, and according to the measurement results, when the irradiation intensity of the lamp head was 400 W/m^2^, the final irradiation intensity on the 24-well plate was 22.8 W/m^2^. The cells treated with UV were irradiated in the 24-well plate, and then, the cells were pipetted evenly and then transferred to the 96-well plate. Each 24-well was repeated 3 times to reduce errors.

### 2.2. Cell Viability Assay

Cell viability was detected by a Cell Counting Kit-8 (CCK-8) (Dojindo Molecular Technologies, Inc., Kyushu, Japan) assays. HL-60 cells were plated in a 96-well plate at a density of 1 × 10^4^ cells per well. The cells were treated with cobalt chloride and/or ultraviolet light. Then, CCK-8 solution was added to each well and the absorbance (OD) values at 450 nm were measured by a microplate reader (Multiskan FC; Thermo Fisher Scientific, Waltham, MA, USA).

### 2.3. Flow Cytometry

For analysis of apoptosis, cells were stained with annexin V-fluorescein isothiocyanate (FITC)/propidium iodide (PI) with an annexin V-FITC apoptosis detection kit (BD Biosciences, Bedford, MA, USA) according to the manufacturer's instructions. The samples were analyzed by FC 500 MPL flow cytometry (Beckman Coulter, CA, USA) within 1 h.

### 2.4. RT-qPCR

Total RNA was isolated from cells using RNAiso Plus (TaKaRa Bio, Shiga, Japan) on ice, and then, the mRNA was reverse transcribed into cDNA using the PrimeScript™ RT kit and gDNA eraser (TaKaRa Bio, Shiga, Japan). A 20 *μ*L reaction system was generated with SYBR® Premix Ex Taq II (TaKaRa Bio, Shiga, Japan), and RT-qPCR was performed according to the standard reaction conditions: an initial denaturation at 95°C for 30 s, followed by 40 cycles at 95°C for 5 s and 60°C for 20 s. The glyceraldehyde 3-phosphate dehydrogenase (GAPDH) was used as an internal reference, and the sequences of the primers (Sangen Biotechnology Shanghai Co., Ltd., Shanghai, China) are shown in Table 1. The results were analyzed using the 2^–ΔΔCq^ method.

### 2.5. Western Blot

Total proteins were collected from cells using RIPA lysis buffer (Solarbio, Beijing, China) with protease inhibitors (Solarbio, Beijing, China) on ice and quantified by a bicinchoninic acid (BCA) assay (Solarbio, Beijing, China). Equal amounts of protein were fractionated by sodium dodecyl sulphate-polyacrylamide gel electrophoresis (SDS-PAGE) and transferred onto polyvinylidene fluoride (PVDF) membranes (Solarbio, Beijing, China). The protein bands were blocked in freshly prepared 5% skim milk, followed by blotting with antibodies against Bcl-2 (1 : 1000; Abcam, Cambridge, UK), Bax (1 : 1000; Abcam, Cambridge, UK), caspase 3 (1 : 1000; Cell Signaling Technology, USA), cleaved-caspase 3 (1 : 1000; Cell Signaling Technology, USA), caspase 9 (1 : 1000; Cell Signaling Technology, USA) and GAPDH (internal reference gene, 1 : 1000; Elabscience Biotechnology Co., Ltd., China) at 4°C overnight. Then, peroxidase-conjugated secondary antibodies (1 : 5000; Elabscience Biotechnology Co., Ltd., China) were used to incubate the membranes. Subsequently, electrochemiluminescence (ECL) solution was applied to detect each band with a chemiluminescence imaging system. The gray value of the protein bands was measured by ImageJ software and normalized to the expression of GAPDH.

### 2.6. Statistical Analysis

Statistical analysis was performed using IBM SPSS V 25 and GraphPad Prism 7. The data were shown as the mean ± standard deviation, and all experiments were repeated three times. Comparisons among groups were performed using one-way ANOVA followed by the Bonferroni correction for multiple pairwise comparisons. *P* < 0.05 was considered a significant difference.

## 3. Results

### 3.1. UV LED and CoCl_2_ Inhibited the Viability of HL-60 Cells

**Figure 1:**
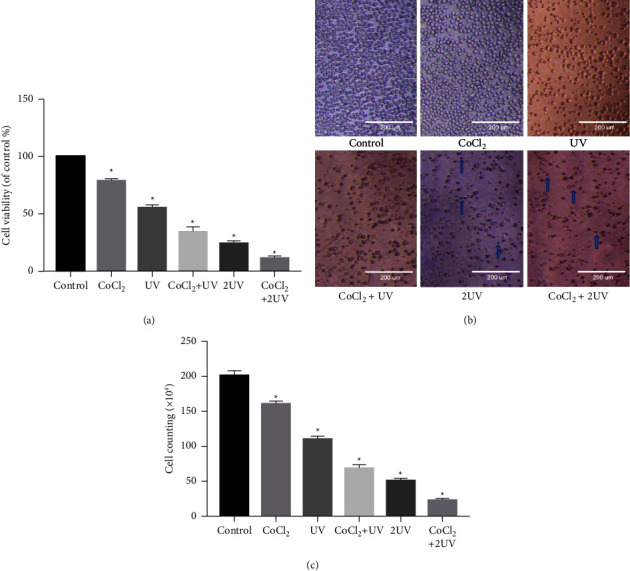
Effects of UV LED and hypoxia on the viability of HL-60 cells. (a) UV LED and CoCl_2_ restrained the vitality of HL-60 cells. (b) Morphological characteristics of HL-60 cells in different groups. Magnification ×100. (c) Microscopic counting of the cells in different groups after 48 hours of culture. Dead cells and lysed cytoplasmic bodies were indicated by arrows. ^∗^*P* < 0.05 vs. the control.

We found that UV and CoCl_2_ reduced the viability of HL-60 cells. The cell viability ranged from high to low as follows: CoCl_2_ group, UV group, CoCl_2_ + UV group, 2UV group, and CoCl_2_ + 2UV group (Figure 1(a)). These results indicated that ultraviolet radiation had a significant effect on inhibiting cell viability under hypoxia.

Microscopic examination showed that the cells in the control group were arranged neatly, round and transparent, with a complete morphology and high cell density. However, in the treatment group, the cells had a disorderly arrangement, the shrinkage and transparency decreased, and the number of cells was significantly lower than that in the control group. A certain proportion of dead cells and lysed cytoplasmic bodies were observed in the 2UV group. The cells showed the weakest growth in the 2UV + CoCl_2_ group, and a large number of dissolved cells were observed (Figures 1(b) and 1(c)).

### 3.2. UV LED and CoCl_2_ Induced Apoptotic and Necrotic Death of HL-60 Cells

**Figure 2:**
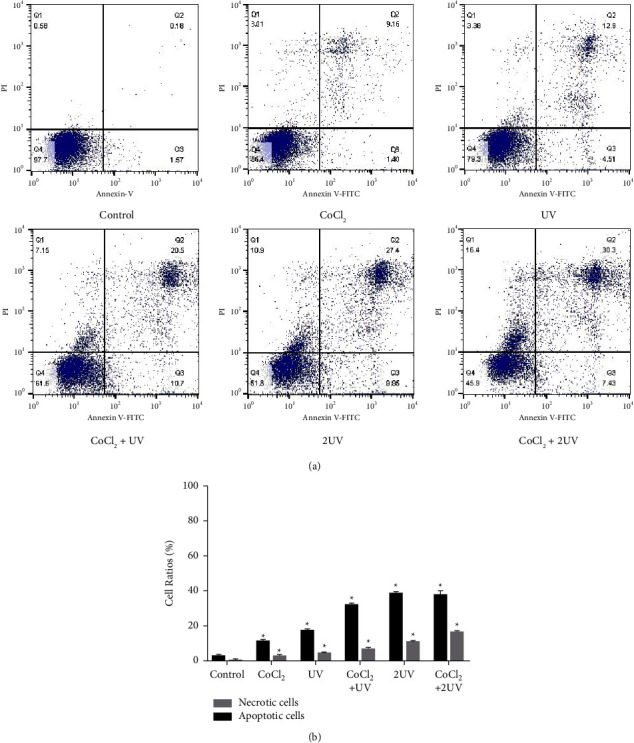
Effects of UV LED and hypoxia on HL-60 cell apoptosis. (a) The apoptosis of the cells exposed to UV LED and CoCl_2_ in different groups was detected by flow cytometry. The four quadrants were as follows: lower left, viable cells; lower right, early apoptotic cells; upper right, late apoptotic cells; and upper left, necrotic cells. (b) The percentages of total apoptotic cells and necrotic cells exposed to UV LED and CoCl_2_. ^∗^*P* < 0.05 vs. the untreated cells.

We then observed apoptosis in the HL-60 cells treated with ultraviolet light and CoCl_2_. As expected, ultraviolet light and CoCl_2_ treatment induced apoptosis of cells. When cells were exposed to both UV and CoCl_2_, the rate of apoptotic cells was higher than that of either alone. The ratio of apoptotic cells also increased with increasing UV irradiation times. However, when the UV times were increased and CoCl_2_ was added, the ratio of both apoptotic and necrotic cells increased significantly, indicating that 2UV + CoCl_2_ mainly induced apoptosis and necrosis (Figure 2).

### 3.3. Changes in Bax, Bcl-2, and Caspase 3 mRNA Levels

**Figure 3:**
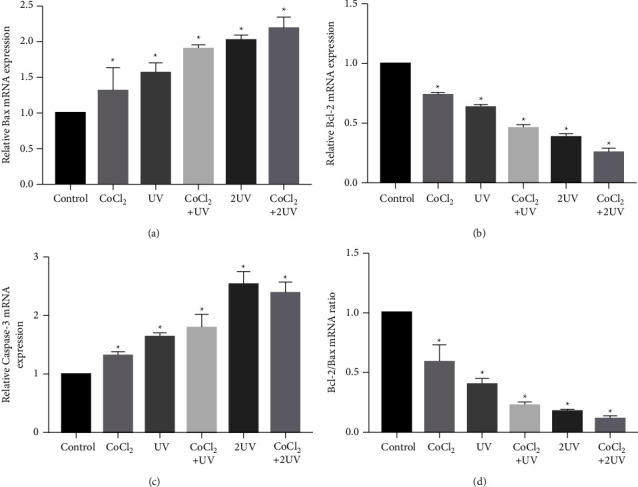
UV LED irradiation and hypoxia reduced mRNA expression of Bcl-2 and increased mRNA expression of Bax and caspase 3. The relative mRNA expression levels of Bcl-2 (a), Bax (b) and caspase 3 (c) in the cells exposed to UV LED and CoCl_2_ in different groups were compared with those of the untreated cells (defined as 1). (d) The Bcl-2/Bax mRNA ratio in different groups. ^∗^*P* < 0.05 vs. the control.

To confirm the apoptosis of cells treated by ultraviolet and hypoxia, we detected the expression levels of Bcl-2, Bax, and caspase 3 mRNA using RT-qPCR. We observed that the mRNA expression of Bcl-2 was substantially lower in the CoCl_2_ group, UV group, CoCl_2_ + UV group, 2UV group, and CoCl_2_ + 2UV group than in the control group, but the expression of Bax and caspase 3 increased significantly. The expression of caspase 3 increased in ascending order in the CoCl_2_ group, UV group, CoCl_2_ + UV group, and 2UV group but was higher than that in the CoCl_2_ + 2UV group. The ratio of Bcl-2/Bax also decreased with the change in exposure (Figure 3).

### 3.4. Evaluation of Bax, Bcl-2, Caspase 3, and Caspase 9 Protein Levels

**Figure 4:**
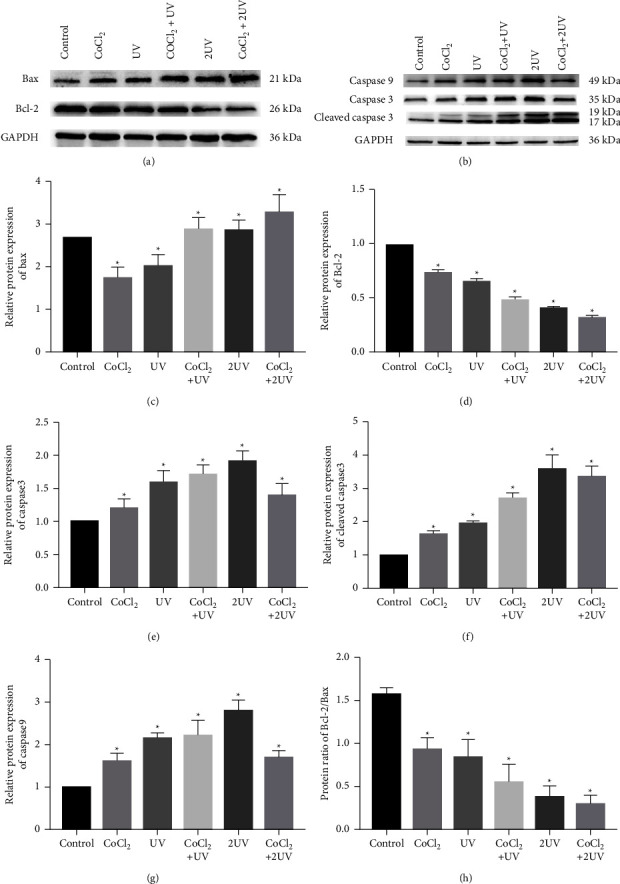
UV LED irradiation and hypoxia reduced Bcl-2 protein level and increased Bax, caspase 3, cleaved-caspase 3 and caspase 9 protein levels. (a) Western blot analysis of protein levels of Bax and Bcl-2. (b) Western blot analysis of protein levels of caspase 3, cleaved-caspase 3 and caspase 9. The relative protein levels of Bax (c), Bcl-2 (d), caspase 3(e), cleaved-caspase 3 (f) and caspase 9 (g) in the cells exposed to UV LED and CoCl_2_ in different groups were compared with those of the untreated cells (set as 1). (h) The Bcl-2/Bax protein ratio in different groups. ^∗^*P* < 0.05 vs. the control.

Then, we continued to detect the expression level of apoptotic proteins by Western blotting. CoCl_2_ and UV significantly increased the expression of Bax, caspase 3, cleaved-caspase 3, and caspase 9 and reduced the expression of Bcl-2 protein compared with untreated HL-60 cells. Therefore, the Bcl-2/Bax ratio decreased with increasing UV irradiation, with or without hypoxic conditions (Figures 4(a), 4(c)–4(e)). Moreover, the protein levels of caspase 3, cleaved-caspase 3 and caspase 9 increased gradually in the CoCl_2_ group, UV group, CoCl_2_+ UV group, and 2UV group, but decreased in the CoCl_2_ + 2UV group (Figures 4(b), 4(f)–4(h)).

## 4. Discussion

UV is a potent inducer of apoptosis and participates in multiple molecular pathways. UV irradiation induces DNA damage to effectively block replication and transcription [11, 12]. DNA damage can lead to the activation of p53; promote the transcription of DNA repair enzymes and antiapoptotic genes, and arrest the cell cycle in the G1 phase. However, when DNA damage cannot be fully repaired, p53 triggers apoptosis and prevents damaged DNA from inducing tumorigenesis [13, 14]. UV can induce the aggregation of the death receptors TNF and Fas in a ligand-independent manner and participate in apoptosis [15]. UV can also stimulate the production of ROS, RNS, and free radicals to promote apoptosis [16]. In recent years, LED UV has received extensive attention. The 280 nm LED UV is located near the strongest UV absorption peak of DNA and nucleoprotein and has a strong killing effect [17]. Our previous study confirmed that LED UV irradiation can inhibit the proliferation of HL-60 cells and induce cell apoptosis and necrosis [10]. UV LED can induce cell cycle arrest in the G0/G1 phase in HL-60 cells [18]. However, the effect of 280 nm LED UV irradiation on HL-60 cells under the hypoxic condition of the bone marrow hematopoietic microenvironment remains to be elucidated.

Oxygen is necessary for tumor cells to maintain cell survival. The activation of the hypoxia-induciblefactor-1 (HIF-1) signaling pathway plays an important role in the response of tumor cells to hypoxia [19]. CoCl_2_ is an iron chelator. As a commonly used chemical hypoxia inducer, CoCl_2_ simulates hypoxia by replacing the iron ions in hemoglobin with cobalt ions. Cobalt chloride can simulate hypoxia to induce apoptosis of different types of cells [20, 21]. In our study, we found that after irradiating HL-60 cells with UV LED and treating HL-60 cells with CoCl_2_ to simulate a hypoxic environment, HL-60 cell growth was significantly inhibited, and apoptosis and necrosis appeared. The apoptotic effects of UV LED on HL-60 cells under hypoxic conditions were stronger than those of single exposure. In addition, the cell apoptosis rate under two rounds of UV LED irradiation was significantly higher than that under single exposure. However, HL-60 cells mainly died when an irradiation dose of 280 nm LED UV was added under hypoxic conditions.

The endogenous mitochondrial pathway and the death receptor pathway are the main signaling pathways of apoptosis. Caspase and Bcl-2 family proteins are involved in both apoptotic pathways [22]. The levels of Bcl-2 and Bax determine the resistance of cells to apoptosis. An elevated Bax/Bcl-2 ratio is associated with apoptosis of cells [23]. In this study, we found an increased Bax/Bcl-2 ratio in cells exposed to CoCl_2_ and UV LED, consistent with the changes in apoptosis rate. Furthermore, we detected the expression of caspase 3/9, and the results showed that CoCl_2_ and UV upregulated the expression of caspase 3/9. However, irradiating cells twice under hypoxic conditions downregulated protein expression levels and are consistent with the necrotic results. This finding indicates that UV LED may induce apoptosis of HL-60 cells by increasing the expression of Bax, inhibiting the expression of Bcl-2, and activating caspase 3/9 under hypoxic conditions. The results suggest that it may be related to mitochondria-mediated apoptosis.

In conclusion, the application of UV LEDs under hypoxic conditions can provide a promising approach to kill residual drug-resistant tumor cells in autologous grafts and may reduce the recurrence rate after autologous hematopoietic stem cell transplantation in leukemia patients.

## Figures and Tables

**Table 1:** The primers for qRT-PCR analysis.

Primer	Sequence (5′ ⟶ 3′)
Bax-forward	AGCGACTGATGTCCCTGTCTCC
Bax-reverse	AGATGGTGAGTGAGGCGGTGAG
Bcl-2-forward	TGCCACCTGTGGTCCACCTG
Bcl-2-reverse	TGGCTGGACATCTCGGCGAAG
Caspase-3-forward	GTGGAGGCCGACTTCTTGTATGC
Caspase-3-reverse	TGGCACAAAGCGACTGGATGAAC
GAPDH-forward	GCACCGTCAAGGCTGAGAAC
GAPDH-reverse	TGGTGAAGACGCCAGTGGA

## Data Availability

The data that support the findings of this study are available from the corresponding authors upon reasonable request.
